# In Response

**DOI:** 10.4269/ajtmh.16-0831b

**Published:** 2017-03-08

**Authors:** Aysegul Taylan Ozkan

**Affiliations:** 1 Department of Medical Microbiology, Faculty of Medicine, Hitit University, Bahçelievler, Turkey. E-mail: aysegultaylanozkan@hitit.edu.tr

Dear Sir:

We thank Pasquale and Tiziana^1^ for their comments on our manuscript entitled “Pediatric visceral leishmaniasis caused by *Leishmania infantum* in Northern Cyprus.”

Indeed the clinical symptoms “typical” for visceral leishmaniasis (VL) are not very specific. We agree with the Pagliano that other clinical signs and conditions should also be taken into account when considering the diagnosis of VL. However, we think that it is of utmost importance to perform good diagnostic procedures, and that clinical suspicion should always be supported by laboratory diagnosis, as in our study. This approach should be adopted by all clinicians working with potential leishmaniasis patients.
